# Xanthan–Polyurethane Conjugates: An Efficient Approach for Drug Delivery

**DOI:** 10.3390/polym16121734

**Published:** 2024-06-19

**Authors:** Narcis Anghel, Iuliana Spiridon, Maria-Valentina Dinu, Stelian Vlad, Mihaela Pertea

**Affiliations:** 1 “Petru Poni” Institute of Macromolecular Chemistry, Gr. Ghica Voda Alley 41A, 700487 Iasi, Romania; spiridon@icmpp.ro (I.S.); vdinu@icmpp.ro (M.-V.D.); vladus@icmpp.ro (S.V.); 2 Department of Plastic Surgery and Reconstructive Microsurgery, ”Sf. Spiridon” Emergency County Hospital Iasi, “Gr. T. Popa” University of Medicine and Pharmacy Iasi, Bulevardul Independentei No. 1, 700115 Iasi, Romania; pertea_mihaela@yahoo.com

**Keywords:** xanthan, polyurethane, drug delivery, ketoconazole, piroxicam

## Abstract

The antifungal agent, ketoconazole, and the anti-inflammatory drug, piroxicam, were incorporated into matrices of xanthan or oleic acid-esterified xanthan (Xn) and polyurethane (PU), to develop topical drug delivery systems. Compared to matrices without bioactive compounds, which only showed a nominal compressive stress of 32.18 kPa (sample xanthan–polyurethane) at a strain of 71.26%, the compressive resilience of the biomaterials increased to nearly 50.04 kPa (sample xanthan–polyurethane–ketoconazole) at a strain of 71.34%. The compressive strength decreased to around 30.67 kPa upon encapsulating a second drug within the xanthan–polyurethane framework (sample xanthan–polyurethane–piroxicam/ketoconazole), while the peak sustainable strain increased to 87.21%. The Weibull model provided the most suitable fit for the drug release kinetics. Unlike the materials based on xanthan–polyurethane, those made with oleic acid-esterified xanthan–polyurethane released the active ingredients more slowly (the release rate constant showed lower values). All the materials demonstrated antimicrobial effectiveness. Furthermore, a higher volume of piroxicam was released from oleic acid-esterified xanthan–polyurethane–piroxicam (64%) as compared to xanthan–polyurethane–piroxicam (44%). Considering these results, materials that include polyurethane and either modified or unmodified xanthan showed promise as topical drug delivery systems for releasing piroxicam and ketoconazole.

## 1. Introduction

Owing to its non-invasive characteristics, simplicity, and adaptability, the transdermal pathway for drug delivery is recognized as the safest and most favored method [1]. Topical delivery films (TDFs) present numerous advantages, such as precise and uncomplicated dosage, enhanced stability, and bioavailability, due to their ability to bypass hepatic metabolism [2,3,4,5]. TDFs are crafted using polymers which not only facilitate the drug release at the junction between the delivery system and the skin but also maintain robust mechanical strength [6,7,8,9]. Depending on the targeted physicochemical and mechanical attributes of the film, a spectrum of natural, synthetic, and semi-synthetic polymers is used to fabricate TDFs. They can be made via varied methods, namely casting, spraying, extrusion, and so on [10,11,12].

Given their biocompatibility, biodegradability, and accessibility, biopolymers like polysaccharides are the core ingredients of topical films [13,14]. Certain polymers such as cellulose [15,16,17], dextran [18], chitosan [19,20,21], alginates [22,23], and xanthan gum [24,25,26,27] are often employed either in their original or modified form or collaborative blends with other polymers (e.g., polyurethanes) for the controlled release of bioactive principles [28,29,30]. Among these, xanthan emerges as the prime choice for utilization in food, cosmetics, and medicinal products.

Materials like xanthan and polyurethanes have been extensively used in drug delivery systems. Nonetheless, despite their widespread use, these biomaterials exhibit significant limitations related to their mechanical properties [31].

Xanthan gum (XG) is a heteropolysaccharide generated by *Xanthomonas campestris* bacteria through submerged aerobic fermentation [32]. Its molecular weight spans from 2 × 10^6^ to 2 × 10^7^ Da [33], comprising a primary chain of repeating D-glucose units linked by β(1-4) linkage, and a side chain of D-mannose and D-glucuronic acid [34]. The helical arrangement of XG’s main chain encircles a side chain via hydrogen bonding, exhibiting unique traits like stability across a broad temperature and pH spectrum, high viscosity at low concentrations, and compatibility with metallic salts [35]. XG finds application in healthcare, like in connective tissue regeneration [36,37], and smart wound dressing materials with controlled rheological properties and drug release rates [38,39], or as delivery systems for biologically active substances [40,41,42,43].

Polyurethanes (PUs) are multifaceted members of high-performance polymeric materials, finding applications in foams, coatings, fibers, adhesives, sealants, electronics, elastomers, actuators, and biomaterials. A rich collection of recent research explores the properties and applications of bio-based PUs as reinforcing materials for intelligent drug delivery carriers [44,45,46,47].

The focal point of the present study is the design of a novel polymeric matrix employing chemically modified xanthan and polyurethane to act as a scaffold for the release, under favorable conditions, of ketoconazole and piroxicam, widely known as antifungal [48,49,50] and anti-inflammatory drugs [51,52,53], respectively. The blend of xanthan’s swelling capacity, polyurethane’s innate mechanical resilience, and the bioactive characteristics of piroxicam and ketoconazole lay a foundation for potential applications, chiefly in the dermato-cosmetic sector. To forge hydrophobic linkages to the polysaccharide chain and prevent XG from forfeiting its structural integrity in humid conditions, functional groups prone to establishing hydrophobic interactions are required [54]. Hence, the objective of this endeavor has been to develop xanthan-based composites by esterifying xanthan with oleic acid for the controlled release of the selected drugs. Polyurethane functions as a binder and plasticizer, its inclusion in the formulation being indispensable to ensure the mechanical robustness of the composite materials.

The novelty in combining these base materials lies in leveraging the complementary properties of xanthan and polyurethane to address the known challenges of rapid degradation and poor mechanical strength found in conventional materials [31,54]. Furthermore, the esterification of xanthan with oleic acid for controlled drug release is a novel approach, enhancing the hydrophobic interactions and thus the structural integrity of the polysaccharide in humid conditions, as supported by recent literature [55,56]. This innovative combination potentially broadens the application spectrum of these biomaterials, particularly in the dermato-cosmetic domain, providing a new avenue for the controlled release of ketoconazole and piroxicam.

## 2. Materials and Methods

### 2.1. Materials

Xanthan (Xn), synthesized by *Xanthomonas campestris*, was received from CP Kelco U.S. The molar ratio of individual units was D-glucose/D-mannose/D-glucuronic/pyruvic acid ketal/O-acetyl = 3.0:3.0:2.0:0.6:1.7, and its molecular weight was approximately 2.5 × 106 Da. 4-Toluenesulfonyl chloride (TsCl), pyridine (Py), methylene chloride, piroxicam, ketoconazole, bovine serum albumin, oleic acid, 4,4′-methylene dicyclohexyl diisocyanate (H12MDI), 4,4′-diphenylmethane diisocyanate (MDI), dimethylol propionic acid (DMPA), polyhexamethylene carbonate diol (PHC–M 2000), 1,4-butanediol (BD), and triethylamine (TEA) were purchased from Sigma-Aldrich (St. Louis, MO, USA). All the materials were used as received, without further purification.

### 2.2. Synthesis of Xanthan Oleate

**Scheme 1:**
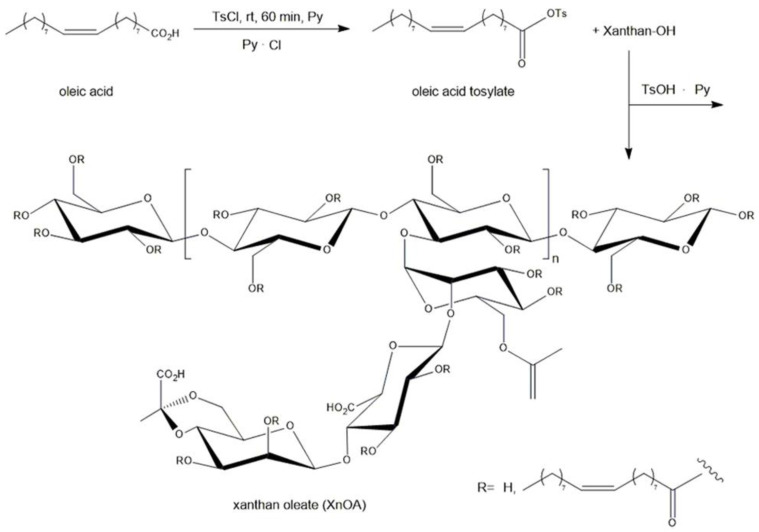
Synthesis of xanthan oleate.

The esterification procedure of xanthan using oleic acid was carried out following the method described by Dimofte et al. [24]. In brief, TsCl (5.1 g), pyridine (6 mL), oleic acid (8.5 mL), and methylene chloride (50 mL) were added to a round-bottomed flask outfitted with a magnetic stirrer, at ambient temperature. After 60 min, xanthan (10 g) was introduced to the mixture in the flask and stirred for 3 h. The product (XnOA) was filtered, rinsed with methylene chloride, water, and ethanol sequentially, and subsequently left to dry at room temperature (Scheme 1).

### 2.3. Synthesis of Polyurethane

**Scheme 2:**
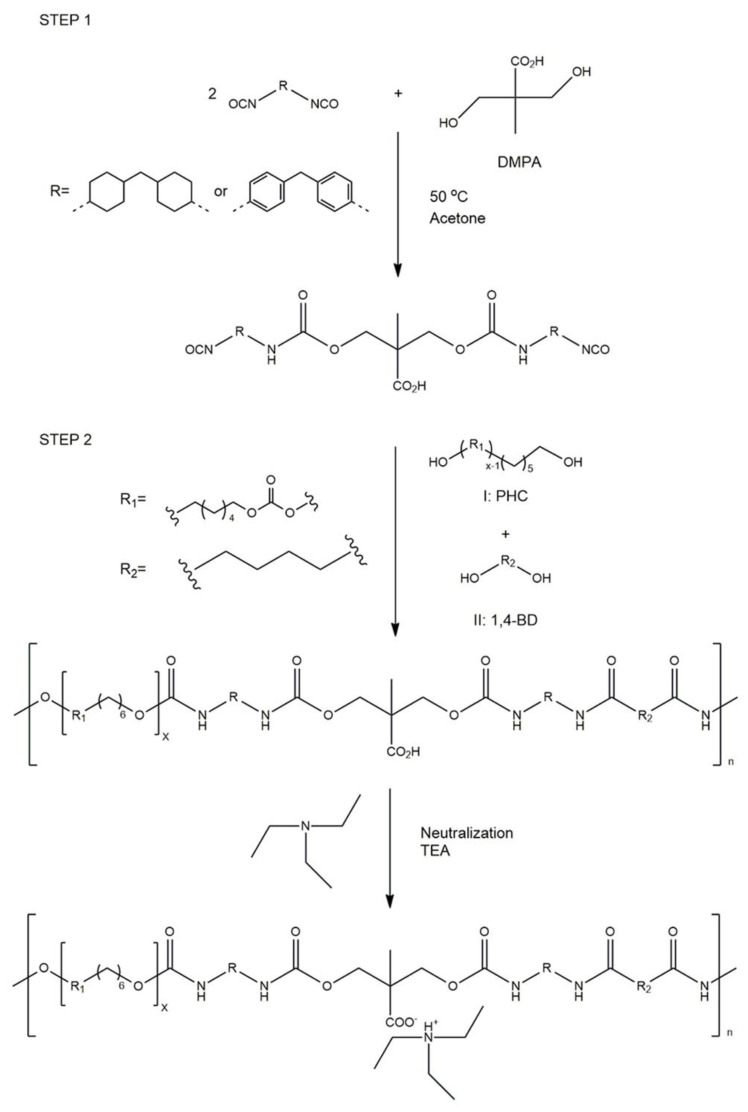
Synthesis pathways of polyurethane water dispersion.

Polyurethane synthesis was carried out as shown in Scheme 2. First, 4,4′-methylene dicyclohexyl diisocyanate (H12MDI—10.48 g) or 4,4′-diphenylmethane diisocyanate (MDI—10 g) alongside 2.64 g of dimethylol propionic acid (DMPA) were stirred in 30 g of acetone (with 99.5 wt% purity) used as a solvent, with the addition of 2–3 drops of dibutyltin dilaurate (DBTL) acting as a catalyst, under reflux conditions (56 °C, 2 h), leading to a homogeneous mixture.

Subsequently, 28 g of polyhexamethylene carbonate diol (PHC–M 2000) was incorporated and stirred for 30 min, followed by the addition of 0.54 g of 1,4-butanediol (BD) serving as a chain extender, with the stirring prolonged for 1 h at 56 °C. Ultimately, the polycarbonate urethane enriched with carboxylic groups was neutralized using 2 g of triethylamine (TEA, 99 wt% purity) for 30 min, and then over 30 min, deionized water (30 g) was gradually added, resulting in the formation of an anionic polyurethane water dispersion.

### 2.4. Preparation of Biomaterials

A quantity of 3 g of either xanthan (Xn) or its modified form (XnOA), along with polyurethane (PU), was mixed at a 1:1 ratio in 300 mL of distilled water, and the blend was heated to 70 °C for 60 min. Upon stirring, this mixture represented the matrix for new materials containing 0.05 g of piroxicam (Xn-PU-P, XnOA-PU-P), 0.05 g of ketoconazole (Xn-PU-K, XnOA-PU-K), and 0.1 g of both piroxicam and ketoconazole (Xn-PU-PK, XnOA-PU-PK), obtained through a series of freeze–thaw cycles, followed by lyophilization.

### 2.5. FTIR (Fourier Transform Infrared Spectroscopy) Analysis

The materials were subjected to FTIR spectroscopy using a Vertex 70 FTIR instrument (Brüker, Billerica, MA, USA) outfitted with an ATR device (ZnSe crystal) set at a 45-degree angle of incidence. Spectral analysis was conducted within the ranges of 4000–600 cm^−1^ and 4500–600 cm^−1^. The measurements employed an average of 64 scans with a spectral resolution of 2 cm^−1^.

### 2.6. NMR (Proton Nuclear Magnetic Resonance) Analysis

NMR spectra, recorded on a Brüker Avance DRX 400 MHz spectrometer, were obtained in deuterated water (D_2_O) and dimethyl sulfoxide solution (DMSO).

### 2.7. Scanning Electron Microscopy (SEM)

SEM images were obtained at a magnification of 200× using a VEGA TESCAN microscope, at an acceleration voltage of 20 kV, at room temperature, with a low-vacuum secondary electron detector.

### 2.8. Mechanical Tests

The mechanical tests were performed at ambient temperature on ethanol-swollen specimens, with dimensions of approximately 8–10 mm in thickness, 10–12 mm in width, and 5–7 mm in height, using a Shimadzu Testing Machine (EZ-LX/EZ-SX Series, Kyoto, Japan). Full contact between the sample surfaces and the compression plates of the testing apparatus was ensured by applying an initial force of 0.1 N before each measurement. The cross-head velocity was set at 1 mm × min^−1^, and the exerted force was maintained at 20 N. The compressive stress (σ, kPa), strain (ε), and elastic modulus (G, kPa) were ascertained following the method previously delineated for other porous materials [27,28,57].

### 2.9. Evaluation of Antimicrobial Activity

The materials’ antimicrobial activity was evaluated using *Salmonella typhimurium* (ATCC 14028), *Staphylococcus aureus* (ATCC 25923), and *Candida albicans* (ATCC 90028) strains, according to the methodology described by Dimofte et al. [24]. Suspensions were obtained from bacterial strains in peptone saline solution with a turbidity of 1° McFarland. A suspension of approximately 1500 UFC (colony-forming units/mL) was obtained through dilution. The surfaces of the examined materials alongside the control sample were inoculated with 10 µL of inoculum of the test strains. The inoculum was extracted using a sterile swab drenched in peptone saline and sowed on the surface of the designated medium after 24 h. The inoculated plates underwent incubation at 37 ± 1 °C for 24 h. Subsequently, the colonies were counted and compared with the control sample.

### 2.10. Evaluation of In Vitro Anti-Inflammatory Activity

The anti-inflammatory activity of materials was evaluated according to the method described by Gunathilake et al. [58] with some modifications. An amount of 100 mg of material comprising P (Xn-PU-P, Xn-PU-PK, XnOA-PU-P, XnOAa-OU-PK) in 5 mL of saline phosphate buffer (PBS, pH = 6.4) and 2 mL of 0.1% bovine albumin solution was incubated at 37 °C for 15 min, and then the mixture was heated at 70 °C for 5 min. After cooling, the absorbance at 660 nm (using PBS solution as blank) was recorded. The solution of bovine albumin was used as a control. Each experiment was conducted in triplicate and the anti-inflammatory activity was calculated as an inhibition percentage by using Equation (1):

(1) $$\%inhibition=100\times \left(1-\frac{A_{s}}{A_{c}}\right)$$

where *A_s_* is the absorption of the sample, and *A_c_* is the absorption of the control.

### 2.11. In Vitro Drug Release

The objective of this experiment was to examine the release patterns of piroxicam and ketoconazole. The materials were weighed and immersed in a 25 mL phosphate buffer solution (PBS, pH = 7.4) maintained at 37 °C. Every 10 min, an equal volume of sample solution (2 mL) was taken and replaced with the same volume of fresh PBS. The absorbance at 254 nm (for ketoconazole) and 285 nm (for piroxicam) was determined using a UV-VIS spectrophotometer. The drug release concentrations were assessed using calibration curves. The tests were conducted in triplicate, and the standard deviation (SD) was calculated.

## 3. Results and Discussions

### 3.1. FTIR Assessment of Xanthan Modified with Oleic Acid

**Figure 1:**
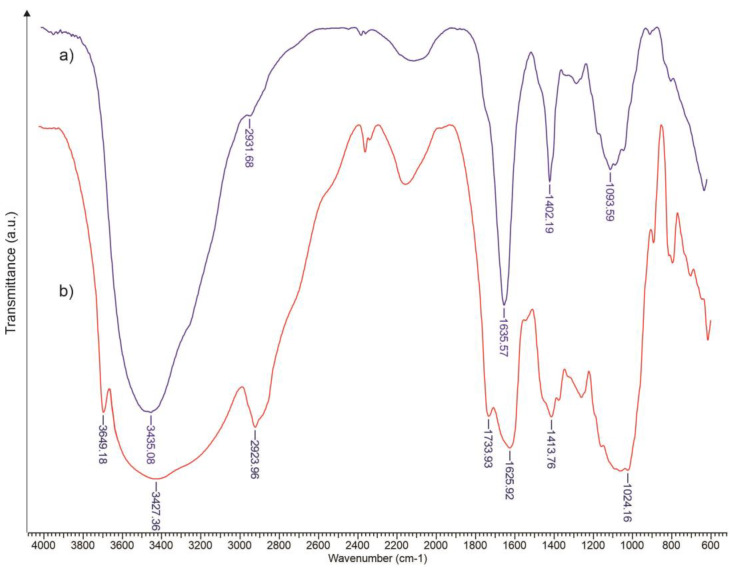
FTIR spectra of xanthan (a) and xanthan esterified with oleic acid (b).

The amplification of the signal at 1402 cm^−1^ and the weakening of the signal at 1253 cm^−1^ in the FTIR spectrum of xanthan (Figure 1a) are attributed to the asymmetric stretching of the carboxylate and the deformation of the C=O group, respectively.

The stretch associated with hydrogen-bonded O–H is visible at 3435 cm^−1^. As depicted in Figure 1b, the hydroxyl groups were detected at 3427 cm^−1^, and the –CH_2_– groups showed stronger vibrations at 2923 cm^−1^ for XnOA. Additionally, a notable absorption at the 1733 cm^−1^, characteristic of the carbonyl group, was identified. The band at 3649 cm^−1^ confirms the double bond in oleic acid.

The esterification of xanthan with oleic acid successfully introduces hydrophobic ester groups, as evidenced by the distinct FTIR bands. The FTIR spectra for the unmodified xanthan and xanthan esterified with oleic acid demonstrate significant differences, particularly in the appearance of the ester carbonyl group band at 1737 cm^−1^ and at 3649 cm^−1^ for the double bond in the modified xanthan. This confirms the successful esterification, enhancing the hydrophobicity of xanthan, which is crucial for its stability in aqueous environments and effective drug encapsulation.

**Figure 2:**
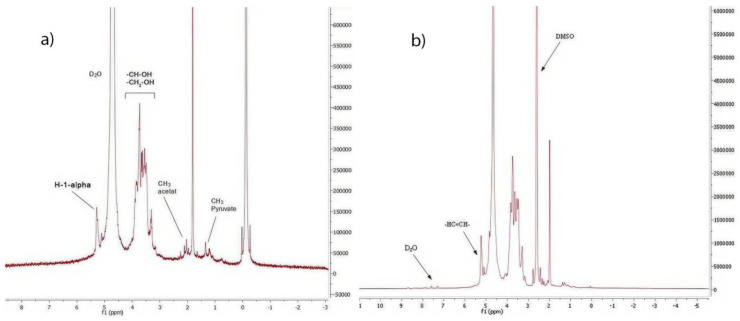
^1^H–NMR spectra for Xa (**a**) and XaAO (**b**).

The ^1^H-NMR spectra for the unmodified and modified xanthan are shown in Figure 2. The peaks at 1.2 and 2.09 ppm are assigned to the methyl group of pyruvate and the methyl group of acetate, respectively. The protons of alcoholic (–CH–OH) and (–CH_2_–OH) are visible via the signals at 3.0–4.0 ppm. The equatorial anomeric proton, H-1-alpha of mannopyranosic unit C, can be observed at 5.2 ppm [59]. The ^1^H–NMR spectrum (Figure 2b) proved the chemical modification of xanthan via esterification with oleic acid. The chemical shift at 5.3 ppm in the XaAO spectra was ascribed to olefinic protons (–HC=CH–) [60].

**Figure 3:**
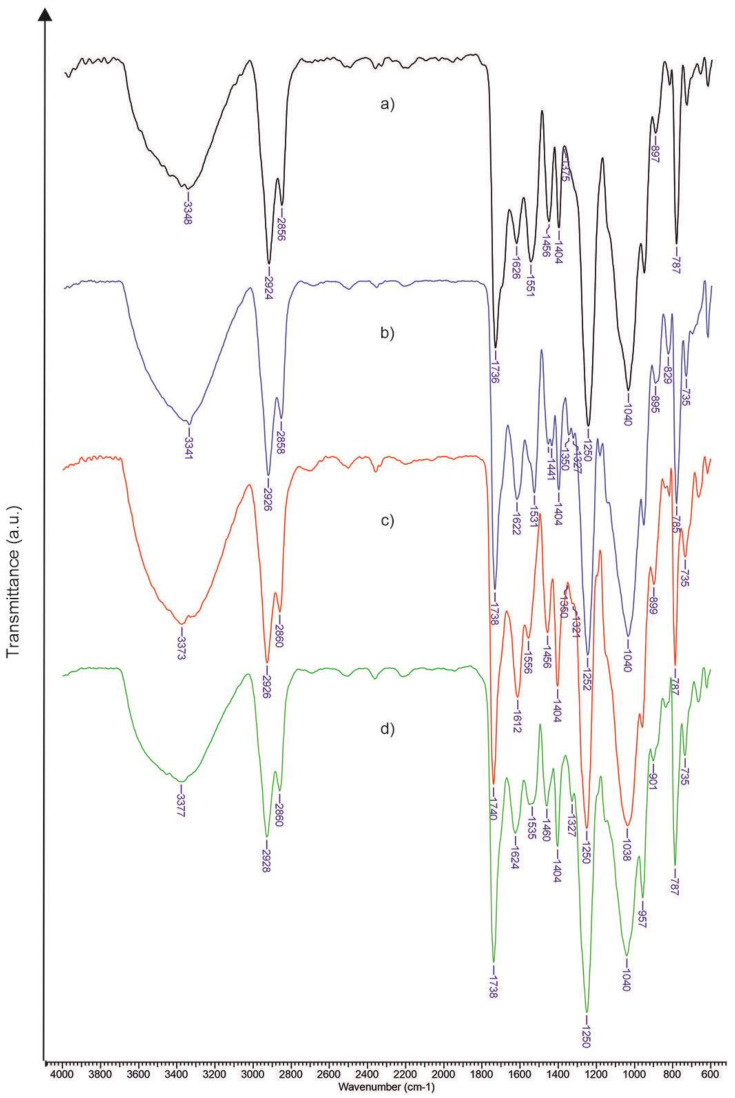
FTIR spectra for materials containing unmodified xanthan: (a) Xn-PU; (b) Xn-PU-P; (c) Xn-PU-K; (d) Xn-PU-P/K.

**Figure 4:**
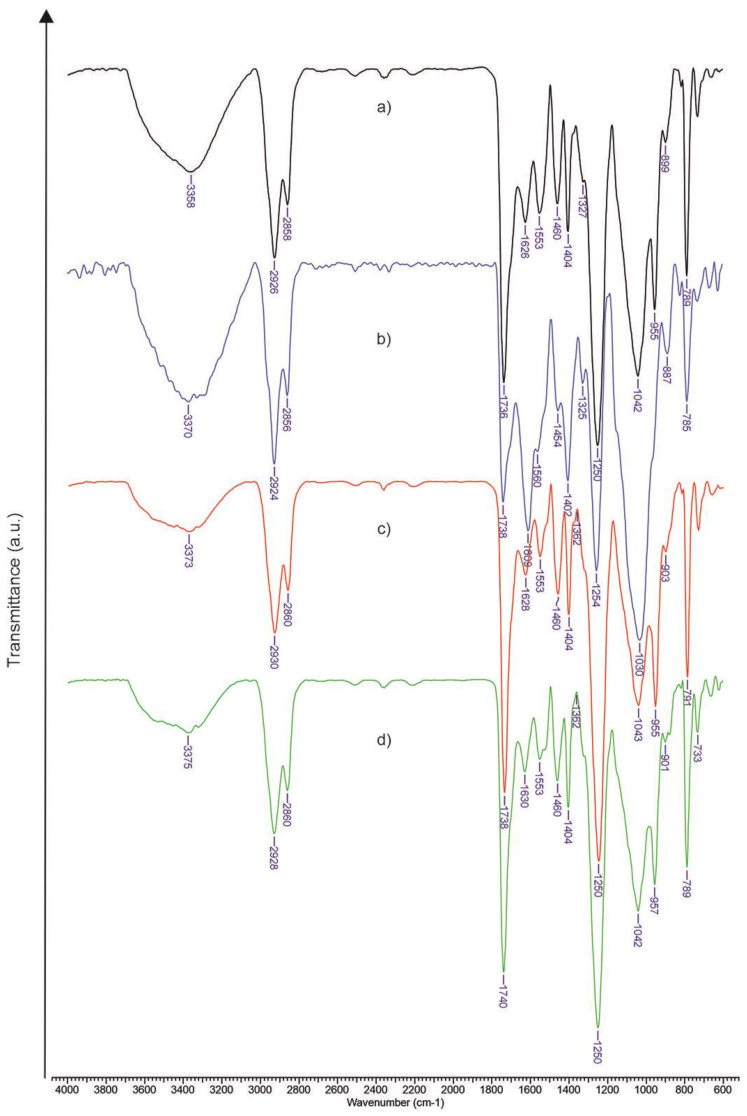
FTIR spectra for materials containing xanthan esterified with oleic acid: (a) XnOA-PU; (b) XnOA-PU-P; (c) XnOA-PU-K; (d) XnOA-PU-P/K.

The FTIR spectra for the synthesized materials are shown in Figure 3 and Figure 4. The band intensity between 3340 and 3377 cm^−1^, associated with the hydroxyl groups, shows an increase while the vibrations related to aliphatic C–H stretching are evident around 2925–2856 cm^−1^. The bands at 1624 and 1458 cm^−1^ are attributed to the asymmetric and symmetric stretching of the carboxylate ions, respectively.

The bands observed at 1180 and 1529 cm^−1^ are linked to amide-II (N–H) bending and S=O symmetric stretching, respectively, confirming the inclusion of piroxicam in the materials. The distinctive bands for ketoconazole appear at 839 and 1238 cm^−1^, associated with the C–N group’s axial stretching and the C–Cl group, respectively.

The FTIR spectra of the drug-loaded composites show characteristic bands for ketoconazole and piroxicam, indicating their successful integration into the polymer matrices. The intensity changes in specific bands suggest alterations in the structural order and interactions within the composites, with modified xanthan showing enhanced crystallinity and structural integrity compared to the unmodified xanthan.

The FTIR analysis confirms the successful incorporation of drugs into both the unmodified and modified xanthan–polyurethane matrices, with the modified xanthan showing improved structural properties. This highlights its potential for more effective drug delivery.

From the obtained spectra, the parameters related to the order level and hydrogen bond strength for the examined materials can be computed. Xanthan possesses crystalline regions with amorphous sections, hinting at a certain structural arrangement. Moreover, the xanthan macromolecules’ crystalline liquid phases remain consistent across a broad concentration spectrum. The formation of these crystalline areas arises from the stable helical structure of the xanthan macromolecule, a result of hydrogen bonding and electrostatic forces. Factors such as the interaction type and intensity between the material components significantly impact these structural features. Hence, aspects like the hydrophilic/hydrophobic nature of the system’s elements, the amorphousness level, hydrogen bond count, and the polar topological surface collectively determine the crystallinity, hydrogen bond strength, and overall structural organization.

The physical attributes of the two active ingredients are detailed in Table 1 [61]. It is highlighted that ketoconazole [62] possesses a semi-crystalline nature, while piroxicam [63] is amorphous.

As proposed by Colomn and Carrillo [64], the total crystalline index (TCI) is determined by the ratio of the absorption bands at 1376 cm^−1^ (C–H bending) to 2902 cm^−1^ (C–H stretching). The formation of hydrogen bonds decreases the intensity of the C–H stretching vibration, which consequently increases the TCI value. The lateral order index (LOI) is computed from the difference in absorbance of the bands at 1437 cm^−1^ (–CH_2_– symmetric bending) and 899 cm^−1^ (C–O–C symmetric in-plane stretching). Pyranose oxygen facilitates the hydrogen bond establishment among the polysaccharide macromolecules with an enhanced order, causing a reduction in the symmetrical in-plane C–O–C vibrations and, thus, a higher LOI value. The hydrogen bond intensity (HBI) is derived by comparing the absorbance bands at 3336 cm^−1^ (–OH stretching) with those at 1336 cm^−1^ (C–OH in-plane bending). This parameter provides insights into chain movement and bond proximity, factors closely associated with crystalline zones, and the consistency of intermolecular arrangements. The TCI, LOI, and HBI values from the FTIR spectra are shown in Table 2.

From the data in Table 2, a clear rise in the crystallinity of the polymer matrix is observed for materials containing chemically modified xanthan, moving from 0.77 to 1.124. This is attributed to the hydrophobic interactions among the hydrocarbon chains of oleic acid, which induce van der Waals bonding forces. These interactions promote the orderly arrangement and packing of the xanthan macromolecules, bringing the chains closer together and making it more likely to establish multiple new hydrogen bonds. The outcome is a rise in crystallinity. With the introduction of drugs, the crystallinity undergoes changes, predictably based on their amorphous or semi-crystalline nature. Introducing piroxicam to the base material leads to a TCI decrease of about 5.2% for the original xanthan and a more significant 27.5% reduction for the xanthan esterified with oleic acid, given piroxicam’s amorphous nature and hydrophilic properties, as indicated in Table 1. In contrast, the addition of ketoconazole, having a semi-crystalline structure, resulted in an increase in the TCI value when compared to the base matrix measurements (34.5% for Xn-PU and 1.2% for XnOA-PU). For the materials containing both drugs, the TCI value lay between those observed for matrices with each drug separately.

Considering the strength of hydrogen bonds and correlating the information in Table 1 and Table 2, the biomaterial with only piroxicam had the highest HBI index due to its larger polar surface (108 Å) and the significant number of atoms involved in forming hydrogen bonds (12), especially when compared to ketoconazole (logP 4.35 and number of hydrogen bond acceptors 6). Data from Table 2 suggest that both the structural organization and the LOI value were more pronounced for the biomaterials only containing ketoconazole, roughly 54% for Xn-PU-K and 62% for XnOA-PU-K, because of its semi-crystalline nature and hydrophobicity, which aligns well with oleic acid residues.

The TCI measures the crystallinity of the polymer matrix. Higher values indicate more ordered and crystalline structures. Xn-PU-K showed the highest TCI (1.175) among the unmodified xanthan samples, indicating that ketoconazole enhances the crystallinity due to its semi-crystalline nature. For the modified xanthan samples, XnOA-PU-K had the highest TCI (1.137), suggesting that ketoconazole also enhances the crystallinity in the oleic acid-modified xanthan. XnOA-PU exhibited higher crystallinity (1.124) than Xn-PU (0.77), showing that xanthan modification with oleic acid increases the overall order in the structure.

HBI provides insights into the strength and number of hydrogen bonds in the material. Higher values indicate stronger hydrogen bonding. Xn-PU-P had the highest HBI (0.893) among the unmodified samples, reflecting piroxicam’s ability to form extensive hydrogen bonds due to its larger polar surface area. Among the modified xanthan samples, XnOA-PU-P showed the highest HBI (1.184), indicating even stronger hydrogen bonding, likely due to the enhanced structural integrity provided by oleic acid. The oleic acid modification generally increased the HBI values, indicating enhanced hydrogen bonding within the matrix.

LOI measures the order within the polymer chains. Higher values signify a higher degree of lateral structure organization. Xn-PU-K and XnOA-PU-K showed the highest LOI values (3.52 and 5.371, respectively), indicating that ketoconazole significantly enhances the lateral order within the matrix. XnOA-PU had a higher LOI (2.024) compared to Xn-PU (1.608), suggesting that oleic acid modification improves the lateral organization of the polymer chains. The oleic acid modification of xanthan significantly enhanced the crystallinity, hydrogen bonding, and lateral order of the polymer matrix.

Ketoconazole, due to its semi-crystalline nature, enhanced the structural organization of both the unmodified and modified xanthan matrices. Piroxicam, with its ability to form hydrogen bonds, contributed to increased hydrogen bond intensity, especially in the modified xanthan matrices. The combined presence of piroxicam and ketoconazole resulted in intermediate values of TCI, HBI, and LOI, indicating a balanced influence of both drugs on the structural properties of the biomaterials.

The chemical modification of xanthan through esterification with oleic acid gave the base matrix a slightly hydrophobic character and an increase in the degree of ordering of the macromolecules (LOI—from 1.608 to 2.024) due to the hydrophobic interactions between the hydrocarbon chains of the unsaturated fatty acid. Concurrently, the enhanced degree of packing of macromolecular chains through hydrophobic interactions reduces the distance between them, thereby increasing the likelihood of forming hydrogen bonds overall (HBI increased from 0.171 to 0.89). The simultaneous presence of the two active principles in both types of biomaterials determines the placement of the TCI, HBI, and LOI index values between the specific reference values for each drug. In other words, instead of primarily establishing hydrogen bonds between the polymer matrix and the active principle, the interactions between the two drugs become dominant.

### 3.2. Mechanical Properties

The uniaxial compressive tests were used to evaluate the elasticity, toughness, and stability of the diverse materials formulated from Xn-PU and XnOA-PU, and loaded with piroxicam (P), ketoconazole (K), or a mix of piroxicam/ketoconazole (P/K).

**Figure 5:**
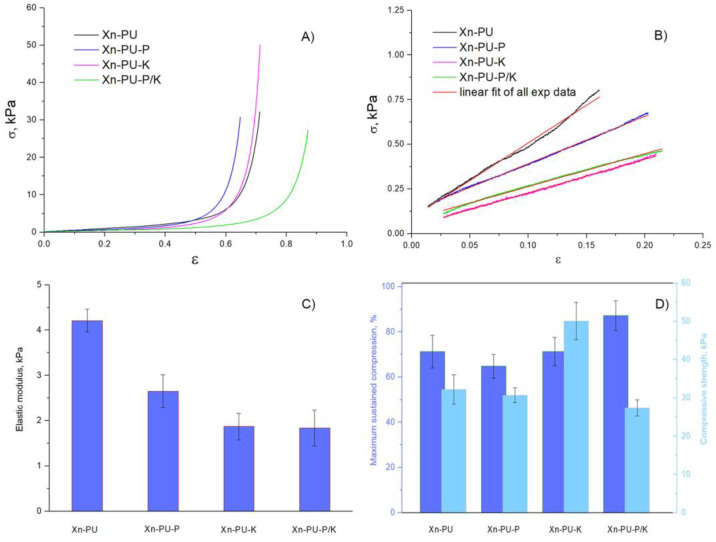
Mechanical properties of swollen Xn-PU-based formulations under compression: (**A**) Stress–strain profiles of Xn-PU-based formulations; (**B**) linear dependence of stress–strain curves; (**C**) elastic modulus calculated according to the standard procedure; (**D**) maximum sustained compression (dark blue color) and compressive strength (light blue color). The standard deviation is presented as error bars.

**Figure 6:**
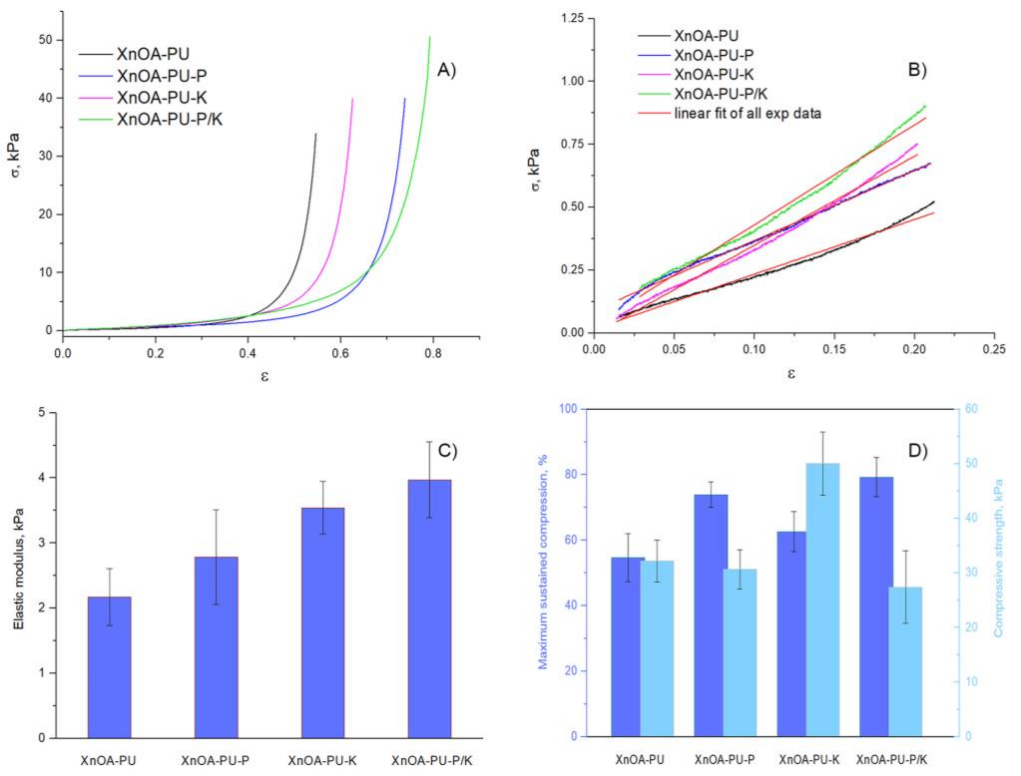
Mechanical properties of swollen XnOA-PU-based formulations under compression: (**A**) Stress–strain profiles of XnOA-PU-based formulations; (**B**) linear dependence of stress–strain curves (**C**) elastic modulus (dark blue color) calculated according to the standard procedure; (**D**) maximum sustained compression (dark blue color) and compressive strength (light blue color). The standard deviation is presented as error bars.

**Figure 7:**
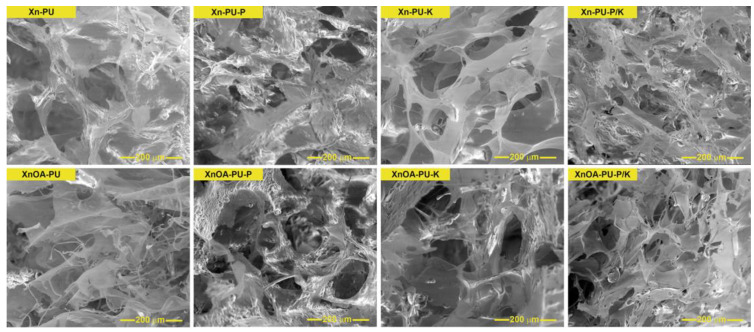
SEM micrographs of biomaterials.

All the biomaterials displayed typical compressive stress–strain (σ–ε) profiles, indicative of macroporous materials (Figure 5A and Figure 6A). Therefore, every formulation, whether containing bioactive compounds (like K, P, K/P) or not, could be compressed beyond 50% strain without inducing any fracture, a phenomenon attributed to the full release of the solvent (ethanol) from the macroporous frameworks of the formulations (refer to the SEM images in Figure 7) upon compression. Analogous outcomes have been documented earlier for the following: (i) xanthan–alginate or oleic acid-altered xanthan–alginate-based formulations [24]; (ii) physically cross-linked chitosan/dextrin cryogels [57]; (iii) chitosan-centric polyelectrolyte complex cryogels [65]; and (iv) silk fibroin cryogels [66].

The elastic modulus (G, kPa) for all the formulations was deduced from the gradient of the linear segment of the stress–strain curves (Figure 5B and Figure 6B), aligning with the protocol previously described [24].

The values for the elastic modulus of Xn-PU- and XnOA-PU-based formulations are depicted in Figure 5C and Figure 6C, while the maximum sustained compression and compressive strength values are showcased in Figure 5D and Figure 6D. It can be seen that the mechanical attributes of Xn-PU- and XnOA-PU-based materials are influenced by the chemical composition of each formulation and by the choice of drug for entrapment: piroxicam—samples Xn-PU-P and XnOA-PU-P, ketoconazole—samples Xn-PU-K and XnOA-PU-K, or the blend of both P and K—samples Xn-PU-P/K and XnOA-PU-P/K.

Upon the encapsulation of ketoconazole into the Xn-PU-based matrix, the compressive strength of the sponges rose to approximately 50.04 kPa (sample Xn-PU-K) at 71.34% strain, as opposed to the sample without the bioactive compound, which displayed a compressive nominal stress of merely 32.18 kPa (sample Xn-PU) at 71.26% strain. When a second drug was entrapped within the Xn-PU biomaterial (sample Xn-PU-P/K), the compressive strength value decreased to about 30.67 kPa, yet the maximum sustained strain increased to 87.21%. This outcome could be correlated with the reduction in pore sizes in the Xn-PU-P/K formulations (refer to the SEM micrographs in Figure 7 and Table 3).

Figure 4 and Figure 5 illustrate the mechanical properties of the swollen Xn-PU- and XnOA-PU-based formulations under compression. The compressive strength and elastic modulus vary based on the chemical composition and drug inclusion. The encapsulation of ketoconazole increases the compressive strength significantly, while dual-drug encapsulation reduces it but increases the maximum strain. This is linked to the pore size variations seen in the SEM images (Figure 7).

Table 3 shows the average pore sizes and pore wall thicknesses for all the sponges. The data reveal that the drug inclusion reduced the pore sizes, affecting the compressive strength and mechanical resilience of the materials.

Following the modification of xanthan with oleic acid, there was a notable increase in the elastic modulus values for the formulations loaded with bioactive compounds (Figure 6C). Supporting this observation, the SEM micrographs of the modified samples exhibited denser and thicker walls in the XnOA-PU-P, XnOA-PU-K, and XnOA-PU-P/K formulations, as opposed to the XnOA-PU sponges, as illustrated in Figure 7 and detailed in Table 3.

The SEM micrographs highlight the structural differences between the materials. The modified samples exhibited denser and thicker walls, correlating with the higher elastic modulus values, suggesting more robust material development upon the modification of xanthan with oleic acid.

To conclude, each sample demonstrated good elasticity and durability, showing no signs of cracks within their structures. These characteristics further endorse their potential use in the field of tissue engineering.

### 3.3. Drug Release Kinetics

The investigation of drug delivery from the polymeric matrix was performed. The significance of using mathematical models for assessing the drug release processes is widely acknowledged. These kinetic models play a pivotal role in elucidating the mechanism of release and in quantifying the key parameters like the release exponent [67].

The Weibull distribution has been employed to examine a variety of mechanisms in heterogeneous process kinetics [66,68], and it has proven to be especially effective for modeling drug release. Ignacio et al. [69] established the Weibull model’s superiority over other statistical models through linear regression analysis. This model exhibited enhanced fitting capabilities with significant accuracy. Crucially, it allowed for the identification of the relationships between its parameters and the release process characteristics, which were in turn associated with the morphological properties of the systems being examined.

**Figure 8:**
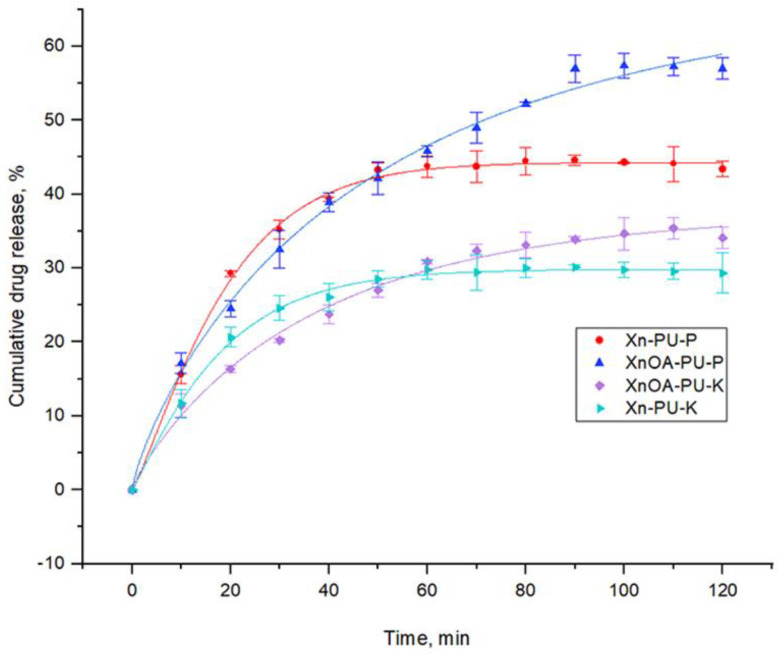
Cumulative drug release over time for biomaterials containing only one bioactive principle.

**Figure 9:**
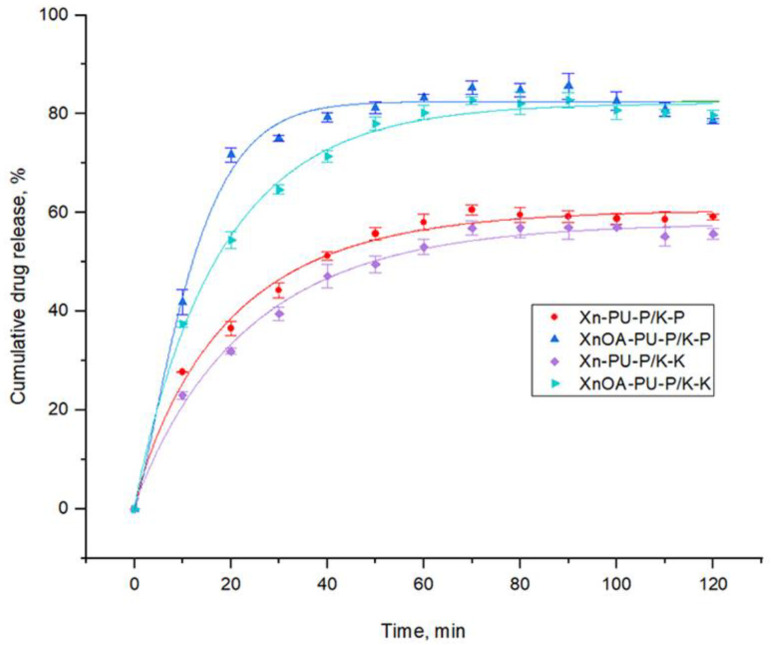
Cumulative drug release over time for biomaterials containing both bioactive principles.

The in vitro drug release patterns are depicted in Figure 8 and Figure 9.

For a comprehensive statistical description of the drug release process, the Weibull cumulative distribution function (CDF), known for its broad utility, was employed. This function provides insights into both straightforward and complex release scenarios. Typically, simpler scenarios involve release mechanisms based solely on diffusion, which can be depicted by either the Higuchi model or the power law. Complex release mechanisms, indicated by higher exponents, often involve combined processes like polymer swelling, matrix erosion, and simultaneous drug diffusion.

The Weibull non-normalized CDF is expressed as:

(2) $$M\left(t\right)=M_{\infty }\times \left(1-e^{-{\left(k\times (t-t_{c}\right)}^{\beta }}\right)$$

where *M_∞_* represents the maximal amount of drug released, *t*—denotes time, *t_c_* is the interpolated time value at the mean of the minimum and maximum amount of bioactive principle released (used for initializing fitting function parameters), and *k* (rate) and *β* (shape factor) are constants linked to specific release mechanisms. The values of these constants have been associated with the diffusion coefficient in matrices characterized by regions of high and low diffusivity.

A shape factor β value of 0.75 or less signifies Fickian diffusion occurring in either fractal or Euclidian spaces. When β lies between 0.75 and 1, the mechanism is a blend of Fickian diffusion and swelling-controlled transport. For β values exceeding 1, the release mechanism becomes more intricate: initially, the release rate rises non-linearly until it reaches an inflection point, after which it declines asymptotically.

Table 4 details the drug release parameters for the examined materials, following the model previously described.

Figure 8 and Figure 9 illustrate that the materials containing both drugs, formulated with the modified xanthan, exhibited higher release rate constants (ranging from 0.055 to 0.078) compared to those with the unmodified xanthan (ranging from 0.042 to 0.05). The release of piroxicam from XnOA-PU-P/K is quicker due to its marginal hydrophilicity, unlike the hydrophobic nature of XnOA. Approximately 82–82.5% of both piroxicam and ketoconazole was released within 120 min from this material. Esterified xanthan slows the release of active ingredients when used individually, due to the hydrophobic interactions between the oleic acid component and the hydrophobic segments of the drugs.

These figures depict the cumulative drug release over time for the biomaterials containing single or both bioactive principles. The Weibull model indicated higher release rates for the modified xanthan matrices, particularly when both drugs were present. The faster release from XnOA-PU matrices might be advantageous for acute treatments.

Ketoconazole, owing to its hydrophobic properties, is more effectively retained by the XnOA-PU polymer matrix compared to Xn-PU. Consequently, the ketoconazole release rate from XnOA-PU was significantly lower (0.027) than that from XN-PU (0.054) as shown in Table 4. Conversely, the presence of slightly hydrophilic piroxicam led to an accelerated release of ketoconazole due to the hydrogen bonding that it forms with ketoconazole. As a result, ketoconazole is released more rapidly (with release rate constants of 0.042 for Xn-PU-P/K and 0.055 for XnOA-PU-P/K). Even when both drugs were used together, while piroxicam may enhance the release of ketoconazole, the latter’s release is still impeded by hydrophobic interactions with the base XnOA-PU matrix.

The shape factor (β) and rate constants (k) provide insights into the release mechanisms. The modified xanthan slows individual drug release due to hydrophobic interactions but facilitates faster release when both drugs are present due to the hydrophilicity of piroxicam aiding ketoconazole release.

**Figure 10:**
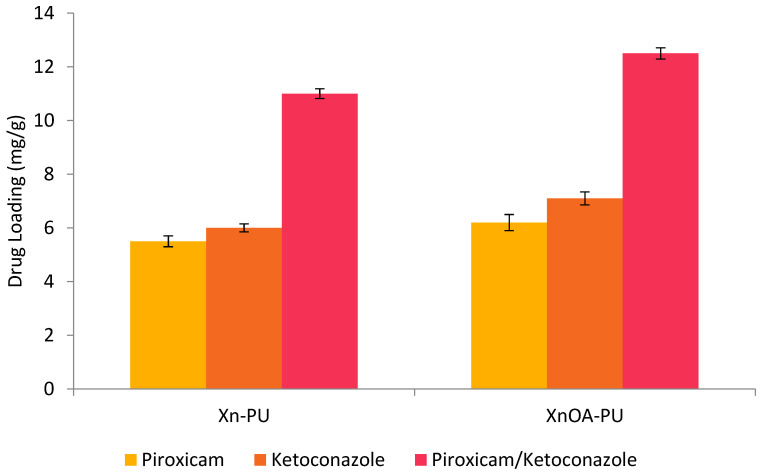
Drug loading capacity of xanthan–polyurethane matrices for piroxicam, ketoconazole, and a combination of both.

**Figure 11:**
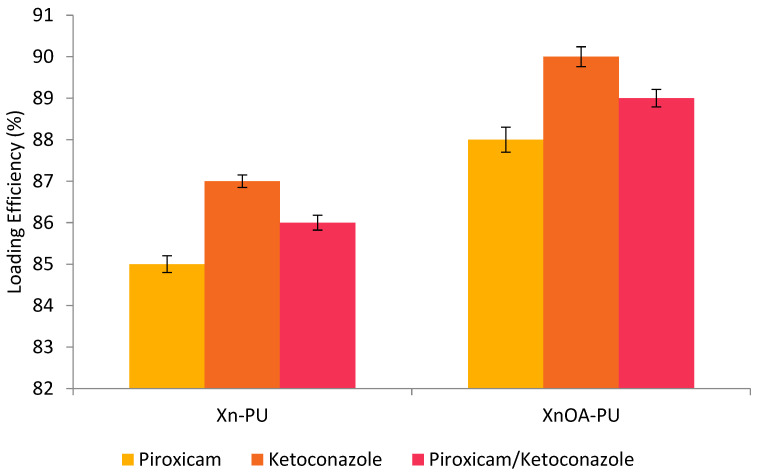
Drug loading efficiency of xanthan–polyurethane matrices for piroxicam, ketoconazole, and a combination of both.

The drug loading capacity and efficiency were evaluated to understand how much of each drug could be incorporated into the xanthan–polyurethane matrices. Figure 10 and Figure 11 show the loading capacity and efficiency of the matrices for piroxicam, ketoconazole, and a combination of both.

The loading capacity of the matrices was assessed by measuring the amount of drug initially added and the amount encapsulated within the matrices. The results indicate that the xanthan–polyurethane matrices could effectively load significant amounts of both drugs, with a slightly higher loading efficiency observed for ketoconazole compared to piroxicam. The dual drug-loaded matrices also showed good loading capacities, demonstrating the potential of these materials for combination drug therapy.

### 3.4. Antimicrobial Activity

*Salmonella typhymurium* and *Staphylococcus aureus* were selected to represent Gram-negative and Gram-positive bacteria, respectively. *Salmonella typhymurium* is a known cause of gastrointestinal ailments, whereas *Staphylococcus aureus* is commonly associated with clinical infections, including those affecting bones, skin, and soft tissues. *Candida albicans*, an opportunistic pathogenic fungus found in the human gut flora, is capable of triggering serious health issues, such as superficial skin and mucosal infections.

The materials under investigation exhibited similar biocidal effectiveness against all the tested bacterial strains (as shown in Figure S1). This consistency suggests effective drug molecule diffusion into the medium, impacting the permeability of bacterial cell membranes.

It is a well-established fact that hydrophilicity plays a significant role in the colonization of microorganisms on material surfaces. The findings of this study highlight the superior antimicrobial activity of materials containing esterified xanthan against the bacterial strains tested. Furthermore, the efficacy of esterified xanthan in hindering fungal growth was also demonstrated.

Figure S1 shows the antimicrobial activity of the tested materials against different strains. The materials containing esterified xanthan exhibited superior antimicrobial effectiveness, likely due to the enhanced drug molecule diffusion and material hydrophilicity impacting the microbial colonization.

### 3.5. Evaluation of Anti-Inflammatory Properties

**Figure 12:**
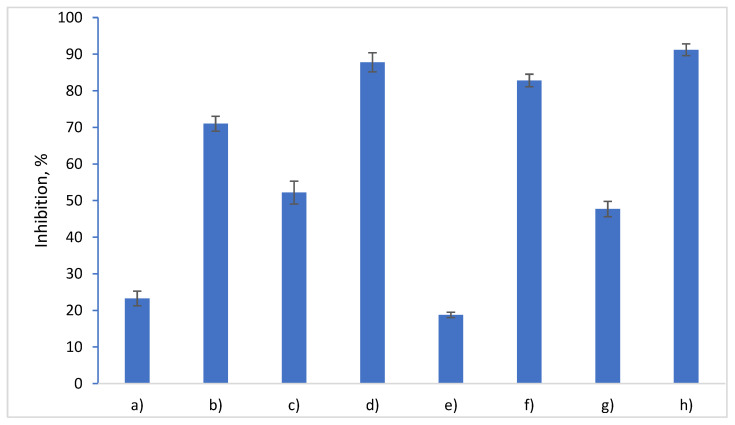
In vitro anti-inflammatory activity of the tested materials: (a) Xn-PU; (b) Xn-PU-P; (c) Xn-PU-K; (d) Xn-PU-P/K; (e) XnOA-PU; (f) XnOA-PU-P; (g) XnOA-PU-K; (h) XnOA-PU-P/K.

The anti-inflammatory potential of the materials is primarily attributed to the inclusion of piroxicam in their composition. The observed inhibition of protein denaturation, specifically bovine albumin, by these materials ranged between 71% and 83% (Figure 12). The denaturation process involves changes in electrostatic, hydrogen, hydrophobic, and disulfide bonding.

Ketoconazole also possesses anti-inflammatory properties [70], contributing to the higher inhibition value observed in Xn/XnOA-PU-P/K compared to Xn/XnOA-PU-P. Piroxicam’s slightly hydrophilic nature (LogP = 3.06) and its larger polar surface area (as detailed in Table 1) facilitate its release from the slightly hydrophobic polymer matrix that includes modified xanthan. This is corroborated by the data presented in Figure 8 and Figure 9, which show that a greater quantity of piroxicam is released per unit time from XnOA-PU-P (64%) compared to Xn-PU-P (44%). This indicates a more marked anti-inflammatory effect in the former system (82.8% inhibition), as opposed to the latter (71% inhibition).

When both drugs are combined, the dynamics change. Ketoconazole, being more efficiently retained by the xanthan esterified with oleic acid due to the hydrophobic interactions, interestingly forms hydrogen bonds with piroxicam. These interactions lead to a delayed release of piroxicam in the external environment, indicating a nuanced interplay between the two drugs within the system. Figure 12 shows the in vitro anti-inflammatory activity of the tested materials. The inclusion of piroxicam significantly inhibited protein denaturation, with modified xanthan matrices showing higher inhibition percentages. This suggests a more potent anti-inflammatory effect due to the better piroxicam release dynamics from the slightly hydrophobic matrices.

## 4. Conclusions

This research successfully developed and characterized novel drug delivery systems consisting of polyurethane and xanthan/modified xanthan. The chemical modification of xanthan with oleic acid was confirmed through FTIR analysis. Drug encapsulation notably influenced the mechanical properties of the Xn-PU and XnOA-PU matrices. For instance, the biomaterials exhibited increased compressive strength (about 50.04 kPa for Xn-PU-K at 71.34% strain) as compared to the samples without bioactive compounds, which showed a compressive nominal stress of only 32.18 kPa (sample Xn-PU) at 71.26% strain. However, the compressive strength decreased to approximately 30.67 kPa with a dual drug entrapped in the Xn-PU matrix (sample Xn-PU-P/K), and the maximum strain sustained rose to 87.21%, possibly due to the reduced pore sizes in these formulations.

The drug release kinetics, modeled effectively using the Weibull distribution, indicated that the matrices with chemically modified xanthan (XnOA-PU) released active ingredients at a 50% slower rate than those with unmodified xanthan for ketoconazole, which could be beneficial for sustained drug delivery. For dual drug formulations, however, the release rate from XnOA-PU matrices was higher, with piroxicam showing a release rate increase of 56% and ketoconazole showing one of 31% compared to the unmodified matrices. This suggests a quicker initial release which might be effective for acute conditions.

The anti-inflammatory activity of the materials, particularly those containing piroxicam, was significantly enhanced in the XnOA-PU matrices. These matrices demonstrated approximately a 16.6% higher inhibition of protein denaturation compared to Xn-PU matrices, indicating a more potent anti-inflammatory effect.

Furthermore, the XnOA-PU matrices exhibited superior antimicrobial activity, underscoring their potential to prevent microbial infections, which is crucial for topical applications. The enhanced performance of the oleic-acid modified xanthan gum in these biomaterials highlights its utility in medical applications requiring sustained drug release and robust antimicrobial protection.

Overall, the novel polyurethane and xanthan-based materials, particularly those modified with oleic acid, showed superior efficacy in controlled drug release, enhanced anti-inflammatory response, and improved antimicrobial activity. These characteristics make the XnOA-PU matrices particularly suitable for topical drug delivery applications, offering the potential for enhanced therapeutic outcomes in treating chronic conditions and acute infections.

This study’s findings suggest that the tailored modification of biopolymers like xanthan gum can significantly enhance the functional properties of drug delivery systems, thereby broadening the scope of their application in dermatological and transdermal therapies.

## Figures and Tables

**Table 1:** Physical properties of ketoconazole and piroxicam.

Physical Property		Ketoconazole	Piroxicam
Hydrogen bond	Donor	0	6
	Acceptor	6	6
LogP, octanol/water partition coefficient		4.35	3.06
Topological polar surface area, Å		69.1	108

**Table 2:** The values of TCI, LOI, and HBI based on FTIR spectra.

Sample	TCI, (A_1376_/A_2902_)	HBI, (A_3336_/A_1336_)	LOI, (A_1437_/A_899_)
Xn-PU	0.77	0.171	1.608
Xn-PU-P	0.73	0.893	1.503
Xn-PU-K	1.175	0.229	3.52
Xn-PU-P/K	1.075	0.821	2.01
XnOA-PU	1.124	0.89	2.024
XnOA-PU-P	0.815	1.184	0.406
XnOA-PU-K	1.137	1.122	5.371
XnOA-PU-P/K	0.789	1.071	1.733

**Table 3:** Average pore sizes and pore wall thicknesses for all the sponges determined from the SEM micrographs with ImageJ 1.41o.

Sample’s Code	Average Pore Size, µm	Average Pore Wall Thickness, µm
Xn-PU	110.03 ± 18.55	42.47 ± 4.79
Xn-PU-P	51.17 ± 17.77	14.22 ± 2.12
Xn-PU-K	58.43 ± 12.03	6.54 ± 1.69
Xn-PU-P/K	39.44 ± 13.48	23.84 ± 4.03
XnAO-PU	50.35 ± 8.23	5.82 ± 1.31
XnAO-PU-P	72.04 ± 17.54	27.37 ± 4.46
XnAO-PU-K	74.71 ± 20.74	22.83 ± 5.31
XnAO-PU-P/K	50.19 ± 13.02	19.62 ± 2.86

**Table 4:** Weibull’s kinetic parameters for drug release experiments.

Sample	*M_∞_*, %	*β*	*k*, min^−1^	Correlation Coefficient, R^2^
Xn-PU-P	44.290 ± 0.323	1.207 ± 0.218	0.051 ± 0.009	0.99813
XnOA-PU-P	67.322 ± 4.798	0.823 ± 0.074	0.020 ± 0.003	0.99557
Xn-PU-K	29.837 ± 0.250	1.109 ± 0.137	0.054 ± 0.006	0.9973
XnOA-PU-K	37.707 ± 1.822	0.902 ± 0.094	0.027 ± 0.003	0.99323
Xn-PU-P/K-K	57.920 ± 1.332	0.942 ± 0.094	0.042 ± 0.002	0.99253
XnOA-PU-P/K-K	82.216 ± 1.003	0.958 ± 0.074	0.055 ± 0.002	0.9956
Xn-PU-P/K-P	60.567 ± 1.237	0.897 ± 0.093	0.050 ± 0.003	0.99216
XnOA-PU-P/K-P	82.498 ± 1.030	1.267 ± 1.175	0.078 ± 0.090	0.99032

## Data Availability

The original contributions presented in the study are included in the article/Supplementary Materials, further inquiries can be directed to the corresponding author.

## Supplementary Materials

The following supporting information can be downloaded at: https://www.mdpi.com/article/10.3390/polym16121734/s1, Figure S1: Antimicrobial activity of the tested materials.
