# Supplementary material for: Xanthan–Polyurethane Conjugates: An Efficient Approach for Drug Delivery
Source: Polymers (Basel). 2024 Jun 19;16(12):1734. doi: 10.3390/polym16121734 (PMC11207320; doi:10.3390/polym16121734)
Supplement: Supplementary file 1 [file polymers-16-01734-s001.zip › polymers-3028445-supplementary.pdf]

Xanthan–Polyurethane Conjugates: An Efficient Approach for Drug Delivery

Narcis Anghel [1,*], Iuliana Spiridon [1], Maria-Valentina Dinu [1], Stelian Vlad [1] and Mihaela Pertea [2]

[1] "Petru Poni" Institute of Macromolecular Chemistry, Gr. Ghica Voda Alley 41A, 700487 Iasi, Romania;
spiridon@icmpp.ro (I.S.); vdinu@icmpp.ro (M.-V.D.); vladus@icmpp.ro (S.V.)

[2] Department of Plastic Surgery and Reconstructive Microsurgery, "Sf. Spiridon" Emergency County
Hospital Iasi, "Gr. T. Popa" University of Medicine and Pharmacy Iasi, Bulevardul Independentei No. 1, 700115 Iasi, Romania; pertea_mihaela@yahoo.com

* Correspondence: anghel.narcis@icmpp.ro

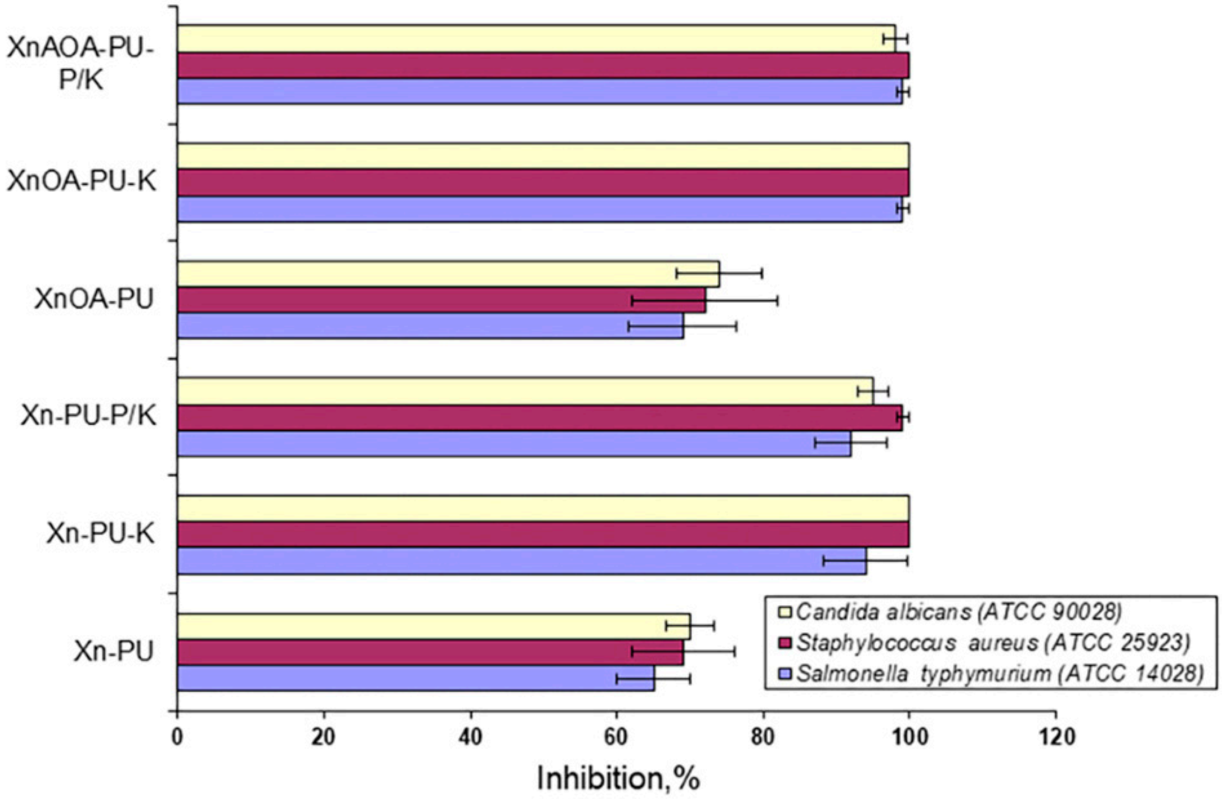

Figure S1. Antimicrobial activity of the tested materials.
